# Silk fibroin/gelatin microcarriers as scaffolds for bone tissue engineering

**DOI:** 10.1016/j.msec.2019.110116

**Published:** 2020-01

**Authors:** Kim A. Luetchford, Julian B. Chaudhuri, Paul A. De Bank

**Affiliations:** a Department of Pharmacy & Pharmacology, University of Bath, Bath, BA2 7AY, UK; b Department of Chemical Engineering, University of Bath, Bath, BA2 7AY, UK

**Keywords:** Microcarriers, Flow focussing, Silk fibroin, Mesenchymal stem cells, Tissue engineering

## Abstract

Microcarrier cell scaffolds have potential as injectable cell delivery vehicles or as building blocks for tissue engineering. The use of small cell carriers allows for a ‘bottom up’ approach to tissue assembly when moulding microparticles into larger structures, which can facilitate the introduction of hierarchy by layering different matrices and cell types, while evenly distributing cells through the structure. In this work, silk fibroin (SF), purified from *Bombyx mori* cocoons, was blended with gelatin (G) to produce materials composed of varying ratios of the two components (SF: G 25:75, 50:50, and 75:25). Cell compatibility to these materials was first confirmed in two-dimensional culture and found to be equivalent to standard tissue culture plastic, and better than SF or G alone. The mechanical properties of the blends were investigated and the blended materials were found to have increased Young's moduli over SF alone. Microcarriers of SF/G blends with defined diameters were generated in a reproducible manner through the use of an axisymmetric flow focussing device, constructed from off-the-shelf parts and fittings. These SF/G microcarriers supported adhesion of rat mesenchymal stem cells with high degrees of efficiency under dynamic culture conditions and, after culturing in osteogenic differentiation medium, cells were shown to have characteristics typical of osteoblasts. This work illustrates that microcarriers composed of SF/G blends are promising building blocks for osteogenic tissue engineering.

## Highlights

• Silk fibroin/gelatin blends were examined as substrates for mesenchymal stem cell growth.
• The presence of gelatin significantly improved cell response.
• Homogeneous silk fibroin/gelatin microcarriers were generated by flow focussing.
• Microcarriers supported mesenchymal stem cell culture.
• Osteogenic differentiation was comparable to that on commercial microcarriers.

## 1 Introduction

Scaffold design for tissue engineering has traditionally relied on a top-down approach, generally attempting to seed cells evenly throughout a relatively large pre-formed scaffold. This has distinct disadvantages; it is often difficult to ensure even cell distribution, and central or core scaffold regions can remain underpopulated. Encapsulating cells within hydrogels is a possible means of overcoming cell distribution issues, but hydrogels have their own shortcomings, such as poor mechanical properties [1]. More recently, bottom-up approaches have been investigated by a number of groups, whereby smaller scaffold fragments or particles are seeded with cells before being moulded or shaped into the final 3D structure [[2], [3], [4]]. In this way, cells are evenly spread throughout the construct, and complexity can be built in by layering different cell or scaffold types. Cell microcarriers, particulate growth matrices with diameters of several hundred microns, are ideal candidates for this type of approach, and also have wider applications in the scale-up of cell production [[5], [6], [7]] and the precise, injectable delivery of therapeutic cells to areas of disease or damage within the body [6,8,9].

Bone tissue is a promising target for the application of microcarriers, either assembled into a construct to treat a sizeable defect, or as injectable cell delivery vehicles to stimulate repair [10]. Microcarriers for applications in bone tissue engineering and repair should possess physicochemical and mechanical properties to support the growth and proliferation of suitable cells, and be able to withstand the mechanical loads present within bone tissue. Silk fibroin (SF), the core protein in silkworm-extruded silk fibres, is a promising biomaterial for use in tissue engineering. It is biocompatible, degrades slowly, can be chemically modified, and can be processed into a wide variety of structures [11,12]. It also has excellent physical properties, being lightweight, strong, highly elastic and thermally stable [11]. Due to its high mechanical strength and positive influence on mineralization, silk is a strong candidate material for bone tissue engineering [13,14]. However, cell adhesion to SF can be poor, so blending with materials that promote cell adhesion can harness the positive attributes of each component material. For example, SF has been blended with other natural polymers including gelatin [[15], [16], [17]], chitosan [18] and hyaluronic acid [19] to improve its properties.

While a wide range of silk-based scaffolds, such as freeze dried sponges [13], nanofibers [20,21] and hydrogels [22,23] have been investigated for bone engineering and repair, there are no reports in the literature regarding the use of spherical silk particles for tissue engineering. Although silk microparticles have been proposed as vehicles for the delivery of drugs and growth factors [[24], [25], [26]], the use of silk fibroin, either alone or blended, for the generation of spherical cell microcarriers has not, to our knowledge, been investigated. This paper describes the first report of silk microcarrier production by microfluidic flow focussing, which generates particles in a reproducible and controllable manner. The device described here was (unlike previously reported systems) assembled entirely from unmodified commercially available fittings, thus facilitating easy adoption and adaptation of this device by other groups [27]. To confirm cell compatibility, SF and SF/gelatin blends (SF/G), were first investigated as two-dimensional films. The mechanical properties of these blends were then evaluated by compression testing to determine the effects of changing the ratio of SF to gelatin. Spherical SF/G microcarriers, produced by flow focussing, were characterized by light and scanning electron microscopy to ascertain their diameter and surface morphology before examining mesenchymal stem cell (MSC) seeding and osteodifferentiation on this particulate scaffold.

## 2 Materials and methods

### 2.1 Materials

Silk cocoons were purchased from Homecrafts Direct (http://www.homecrafts.co.uk/). Foetal bovine serum (FBS) was purchased from Thermo-Fisher. Antibodies for fluorescence-activated cell sorting were obtained from Miltenyi Biotec Ltd., R&D Systems and BD Biosciences as described in Table 1. The osteopontin ELISA kit was purchased from R&D Systems. Upchurch HPLC fittings and connectors described in Section 2.6 were purchased from Kinesis, and glass capillaries were purchased from CM Scientific. All other materials and reagents were purchased from Sigma-Aldrich and used as supplied.

**Table 1:** Antibodies and isotype controls used for the identification and enrichment of rMSCs by FACS.

Antibody	Supplier	Catalogue #	Conjugate	Dilution
Mouse anti-rat CD90.1	Miltenyi Biotec Ltd.	130-102-637	Vioblue	1:11
Mouse anti-rat CD54	R&D Systems	FAB5831F	FITC	1:3.5
Mouse anti-rat CD45	BD Biosciences	561,588	PE-Cy7	1:50
Isotype control mouse IgG2a	Miltenyi Biotec Ltd.	130-098-898	Vioblue	1:11
Isotype control mouse IgG2b	Miltenyi Biotec Ltd.	130-099-119	FITC	1:3.5
Isotype control mouse IgG1κ	BD Biosciences	560,906	PE-Cy7	1:11

### 2.2 Purification of silk fibroin (SF) from silk cocoons

SF was extracted from *Bombyx mori* silk cocoons as previously described [28]. Briefly, chopped cocoons were degummed by boiling in 0.02 M sodium carbonate for 1 h and then washed 5 times in distilled water before being air-dried overnight. The dried silk was then dissolved at 15% *w*/*v* in 9 M lithium bromide by heating at 60 °C for up to 4 h. After cooling to room temperature, the solution was filtered through a 5 μm syringe filter (Sartorius) and dialysed against deionized water using SnakeSkin™ dialysis tubing (3500 Da MWCO, Thermo) until the conductivity of the dialysate did not increase (2–3 days). The SF solution was then either freeze dried (Thermo Savant MicroModulyo) or known volumes were oven-dried overnight to determine the concentration, and the solution stored at 4 °C until required.

### 2.3 Rat mesenchymal stem cell (rMSC) isolation and culture

rMSCs were extracted from the bone marrow of juvenile Wistar rats as described by Zhang and Chan [29]. Cells were initially selected by adherence to tissue culture plastic (TCP) and grown to ~80% confluence prior to enrichment using fluorescence-activated cell sorting (FACS). For this, cells were trypsinized, counted and divided into microcentrifuge tubes containing between 10^5^ and 10^6^ cells per sample. Cells were then pelleted by centrifugation (300 ×*g*, 5 min) and washed in cold FACS buffer consisting of PBS with 1% *v*/v FBS before 30 min incubation on ice. Cells were again pelleted and re-suspended in 50 μL of FACS buffer. The antibodies and isotype controls were added to each sample as appropriate, in the dilutions listed in Table 1. The samples were incubated for 1 h on ice, protected from light. Finally, the samples were washed twice by pelleting and re-suspending in FACS buffer, then finally re-suspended in 500 μL of PBS, at which point FACS was performed using a Becton Dickinson FACSAria III.

The population of cells positively expressing CD90.1 and CD54, and negative for CD45, was cultured in basal medium consisting of αMEM supplemented with 10% foetal calf serum, 2 mM l-glutamine, 100 U/mL penicillin and 100 μg/mL streptomycin. Cells were maintained at 37 °C, in a humidified atmosphere containing 5% CO_2_ and used up to passage 8, after which they lose their capacity to proliferate and differentiate [30].

### 2.4 Formation of silk fibroin/gelatin films

Silk fibroin (SF), gelatin (G) and SF/G blends (SF:G 75:25, 50:50, 25:75) were dissolved in hexafluoroisopropanol (HFIP) at a total protein concentration of 2% *w*/*v* and added to separate wells of multiwell plates at a volume sufficient to coat the base of the well (105 μL/cm^2^). The solvent was evaporated to leave thin protein films, which were crosslinked by treatment with 50 mM 1-ethyl-3-(3-dimethylaminopropyl)carbodiimide hydrochloride (EDC) in methanol for 24 h at 4 °C, which also ensured the transition of SF to the insoluble β–sheet conformation [31]. Wells were then washed three times with dH_2_O, dried in a culture hood overnight, and sterilized by 30 min exposure to UV light (253.7 nm, 0.115 kW, Bioquell). Plates were either seeded with cells immediately or stored at 4 °C until use.

### 2.5 Assessment of cell proliferation and osteogenesis on 2D films

Relative cell metabolism was used as a measure of cell proliferation and was determined by quantifying the reduction of resazurin. To measure cell metabolism by reduction of resazurin to resorufin, rMSCs were seeded in 24 well plates at 2.5 × 10^4^ per well and, at specific time points, subsequently incubated with 0.15 mg/mL resazurin solution at 20% *v*/v of the total culture volume. After 2 h incubation at 37 °C, the fluorescence of the medium was measured at 560/590 nm (excitation/emission) using a BMG Labtech FLUOstar Omega, and cell culture continued in fresh medium.

To promote osteogenic differentiation, rMSCs were seeded in 24 well plates at 3 × 10^4^ per well and grown to confluence in basal medium before switching to osteogenic differentiation medium (ODM), which consisted of basal medium supplemented with 0.1 μM dexamethasone, 0.2 mM ascorbic acid 2-phosphate and 10 mM β-glycerophosphate. Levels of osteopontin (OPN) in cell culture supernatants were subsequently quantified using mouse/rat Osteopontin Quantikine ELISA Kit (R&D Systems) according to the manufacturer's instructions. Prior to this, cell culture medium was completely replaced on day 10 after switching to ODM and supernatants collected on day 14 for OPN analysis. Data were normalized to the number of cells per well, quantified by fluorescent detection of Hoechst-stained cell lysate [32]. Briefly, media was removed from the samples, which were frozen at −80 °C then thawed at 37 °C. Distilled H_2_O was then added to each well before sonicating for 15 min (VWR Ultrasonic Cleaner USC300TH) and then re-freezing. Samples were again thawed at 37 °C, and the resulting supernatants added to an equal volume of 20 μg/mL Hoechst 33342 in TNE buffer (10 mM Tris-HCl, 1 mM EDTA, 2 M NaCl; pH 7.4). A standard curve was constructed using samples with known cell numbers. Fluorescence was read at 350/460 nm (BMG Labtech FLUOstar Omega).

### 2.6 Construction of an ‘off the shelf’ axisymmetric flow focussing device

The flow focussing device was based on a design by Terray & Hart [27] and assembled from a HPLC T-junction (Tee LP PEEK 1/4–28 1/16″ 0.040” Thru Hole) fitted with a central glass capillary (Hollow Round Glass Capillaries ID 0.50 mm OD 0.70 mm). The inner and outer phases were fed into the T-junction through PEEK tubing (ID 0.03″, 1/16” OD) with the output consisting of PEEK tubing with a smaller inner diameter (0.02″). Input tubing was connected to Luer lock syringes *via* tubing-to-cone and cone-to-Luer fittings (Fingertight fitting two-piece PEEK 10–32 Long; Adaptor female luer to 10–32 female PEEK). The two input feeds were controlled by syringe pumps (Harvard Apparatus Pump 11 Plus and Cole Parmer single syringe infusion pump), and consisted of two immiscible fluids, an outer continuous oil phase and an inner aqueous phase.

### 2.7 Microcarrier production by axisymmetric flow focussing

Type A porcine gelatin was dissolved to a concentration of 50 mg/mL in dH_2_O at 60 °C, and SF solution was diluted to the same concentration. Prior to the formation of microcarriers, these solutions were mixed to give blends with SF/G ratios of 100:0, 75:25, 50:50 and 25:75, and then maintained at 60 °C until use. To generate microcarriers, SF or SF/G solution was used as the inner phase in the flow-focussing device, with the inner flow rate (Q_inner_) set at 0.36 mL/h. The outer phase consisted of oleic acid, methanol and Span 80, mixed in a volume ratio of 73:25:2 [33], with the outer flow rate (Q_outer_) set at 7.2 mL/h. The output of the device was collected into a mixture of the outer phase solution diluted 1:1 with methanol and kept on ice. Following filtration, SF microcarriers were washed with PBS, autoclaved in PBS at 121 °C for 15 min and then stored at 4 °C until use. Gelatin-containing microcarriers were cross-linked for 24 h with 50 mM EDC in methanol at 4 °C and then treated in the same way as SF microcarriers.

### 2.8 Morphological analysis of 3D microcarriers

Images of the microcarriers were acquired using a Leica DMI4000B microscope and their diameters then determined using the Particle Analysis function of ImageJ (NIH, Bethesda, MD, USA; http://imagej.nih.gov/ij) [34]. At least 100 particles were analysed for each material type. To analyse their surface topography, microcarriers were freeze-dried, mounted on aluminium stubs, and sputter coated with gold (Edwards Sputter Coater 5150B). Samples were then examined with a JEOL JSM6480LV scanning electron microscope.

### 2.9 Determination of Young's Modulus of SF, SF/G blends and gelatin

To produce discs for compression testing, 1.5 mL aliquots of 5% *w*/*v* solutions of SF, the three SF/G blends and gelatin were added to wells of a 12 well plate. Plates were cooled to 4 °C to gel the gelatin-containing discs, before being frozen at −20 °C. Methanol (1 mL) was added to each well and the discs incubated overnight at 4 °C. After removal of the methanol, SF discs were then thoroughly washed three times with PBS, while SF/G and gelatin discs were further crosslinked by EDC treatment as described in Section 2.4 and then washed in PBS. Once removed from the plates, discs of 13.5 mm diameter and 3–4 mm height were punched out of the solid materials using a cork borer and then examined by uniaxial, unconfined compression analysis using an Instron 5965 testing system at room temperature. All samples were subjected to a loading rate of 0.2 mm/min and measured for 6 min.

### 2.10 Cell seeding and culture on microcarriers

Cells were seeded onto microcarriers in 24 well plates coated with a non-adhesive layer of 1% agarose in DMEM. In a total volume of 500 μL, cells were seeded at a concentration of 3.75 × 10^5^ cells/mL with microcarriers suspended at 2 × 10^3^ particles/mL in basal medium. The plate was incubated on a plate rocker under standard cell culture conditions and, after 24 h, the suspension was filtered through a 70 μm nylon mesh filter to remove unattached cells, which were counted to establish the proportion of cells remaining adhered to the microcarriers. After initial seeding, cell-laden microcarriers were cultured statically in basal medium or transferred into ODM, and cultured for 14 or 28 days.

### 2.11 Analysis of viability and osteodifferentiation on 3D microcarriers

To assess rMSC viability on SF/G microcarriers, cells were seeded as described above and, after 96 h in culture, stained simultaneously with calcein-AM and ethidium homodimer-1 (both 2 μM in PBS) to identify living and dead cells. Cells were washed with PBS, incubated in the staining solution in the dark for 20 min at 37 °C and then examined using a Zeiss LSM510 confocal microscope. To determine osteogenic differentiation of the cells, secretion of OPN was measured as described in Section 2.5 after 14 days in ODM, or alkaline phosphatase (ALP) activity was assayed after 28 days. Microcarriers were washed with PBS, cells were fixed briefly in 4% *w*/*v* paraformaldehyde (maximum of 60 s) and then washed in PBS with 0.05% *v*/v Tween 20. BCIP/NBT solution (5-bromo-4-chloro-3′-indolyphosphate p-toluidine salt and nitro-blue tetrazolium chloride), was then added to the cells for 10 min and the cells washed once more with PBS/0.05% v/v Tween 20. Generation of a dark purple precipitate, indicating the presence of ALP, was assessed using a stereomicroscope (Olympus SZ61TR).

### 2.12 Statistical analysis

Statistical analysis was performed using one-way analysis of variance (ANOVA) with Tukey's honest significant difference post-hoc test using R software [35]. A value of *p* < 0.05 was considered statistically significant.

## 3 Results and discussion

In order to determine the suitability of SF/G blends for use as a substrate for MSC proliferation and osteogenic differentiation, cell-material interactions were initially assessed in 2D by seeding rMSCs on biomaterial films, which were cast within multiwell plates. Suitable blends were then taken forwards to evaluate their ability to form microcarriers which supported MSC adhesion and osteogenesis.

### 3.1 rMSC adhesion to SF and SF/G blends in two-dimensional culture

**Fig. 1:**
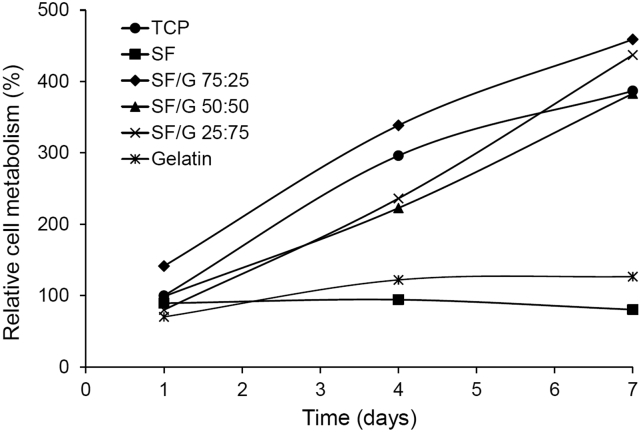
Proliferation of rMSCs on films of SF, gelatin and SF/G blends, measured by reduction of resazurin. Data is presented as the fluorescence signal relative to cells on the untreated TCP control at day 1. Data shown represent the mean value (*n* = 3).

The ability of films of SF, SF/G and gelatin to support rMSC proliferation was assessed over 7 days using the resazurin reduction assay which, unlike a number of other metabolic assays, enables the same population of cells to be examined across multiple time points (Fig. 1). rMSCs proliferated poorly on pure gelatin films, which was unexpected as gelatin is well known as a highly cell-adhesive material and is used routinely in tissue engineering scaffolds [[36], [37], [38]]. In this case, it is possible that the low level of cell adhesion and growth was a result of the gelatin absorbing water from the culture medium and becoming very soft. Swelling of gelatin under physiological conditions is well-established and, although cross-linking reduces the potential for swelling, it does not remove it completely [[38], [39], [40]]. Additionally, it has previously been shown that gelatin-coated substrates softer than 100 Pa did not support the survival of normal fibroblasts, and it appears the same effect may have occurred in this study [41]. SF is less hydrophilic than gelatin, with long regions of hydrophobic amino acid residues in its structure, which organize to form insoluble β-sheets upon exposure to methanol [12,42]. It is thus less likely to absorb water and become destabilized to the same extent as gelatin hydrogels. However, as is apparent from Fig. 1, rMSCs did not proliferate on SF films, while blending SF with gelatin dramatically improved cell growth, with the proliferation of the cells on all three SF/G blends being comparable to growth on the tissue culture plastic (TCP) control. This stark contrast is likely due to the presence of Arg-Gly-Asp (RGD) integrin binding motifs within gelatin [43,44], which are absent from *Bombyx mori* SF [45]. Many studies have examined the use of SF for bone engineering and repair applications, with considerable success. However, even in these studies, cell growth on SF scaffolds is relatively poor, with SF used primarily for its mechanical strength, biodegradability and biocompatibility [13,46,47]. Indeed, there are many examples in the literature of modifications to SF scaffolds in order to improve cell adhesion and growth, including direct chemical modification of SF and blending with other biomaterials or growth factors [20,[48], [49], [50], [51], [52], [53], [54], [55]]. An exception is the use of electrospun SF nanofibres, where improved cell growth on unmodified SF is likely due to the topographical features of the fibres and their mimicry of the dimensions of native ECM [56]. Thus, in the SF/G blends presented in this study, while SF is likely to improve the physical properties of 2D films, the presence of gelatin is required to provide valuable sites for cell adhesion.

### 3.2 Osteogenic differentiation of rMSCs on SF/G films

**Fig. 2:**
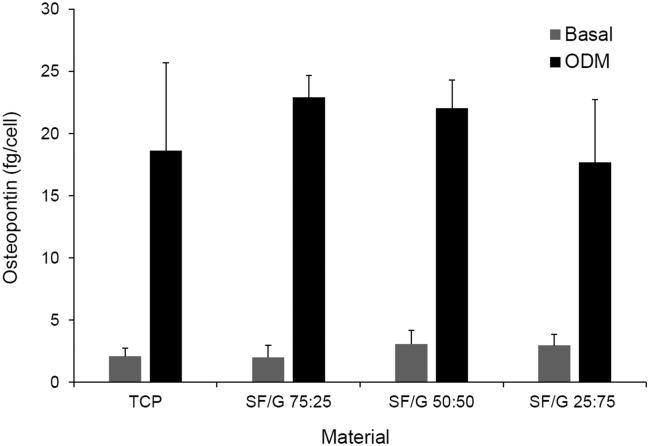
Secretion of osteopontin by rMSCs grown on TCP or SF/G films in basal medium or osteogenic differentiation medium (ODM) for 14 days. Data shown represent mean + standard error (n = 3).

Deposition of calcified ECM is a commonly used measure to determine osteogenic differentiation of MSCs. However, it was found that mineralization stains, such as Alizarin Red [57] and von Kossa [58], gave heavy background staining of SF/G films even in the absence of cells, and so could not be used. Instead, we examined the expression of osteopontin (OPN), a secreted matricellular protein with roles in biomineralization and bone remodelling [59], which is expressed during osteogenesis [60]. Following 14 days culture in either basal medium or osteogenic differentiation medium (ODM), OPN secretion by rMSCs was quantified by an ELISA (Fig. 2). Due to the poor response of rMSCs to SF and gelatin films, these substrates were not examined in these assays.

Cells grown on all three SF/G blends and the TCP control secreted very low quantities of OPN, between 2.08 and 3.08 fg/cell, when cultured in basal medium, with no significant differences observed between the different substrates. However, when cultured in ODM, there was a significant increase in OPN secretion on all surfaces, ranging from 17.68 to 22.92 fg/cell, indicating osteogenic differentiation of the rMSCs on these blended materials. While there was an apparent trend for increasing OPN secretion with increasing SF content in the films, differences in OPN levels between substrates were not statistically significant. It is extremely difficult to compare these relative quantities to similar studies in the literature as data is often reported at the mRNA level and/or as a fold-increase or percentage relative to a control rather than absolute quantity of protein secreted per cell. Nonetheless, it is clear that SF/G blends are capable of supporting the proliferation and osteogenic commitment of rMSCs.

### 3.3 Production of cell microcarriers from SF/G blends

**Fig. 3:**
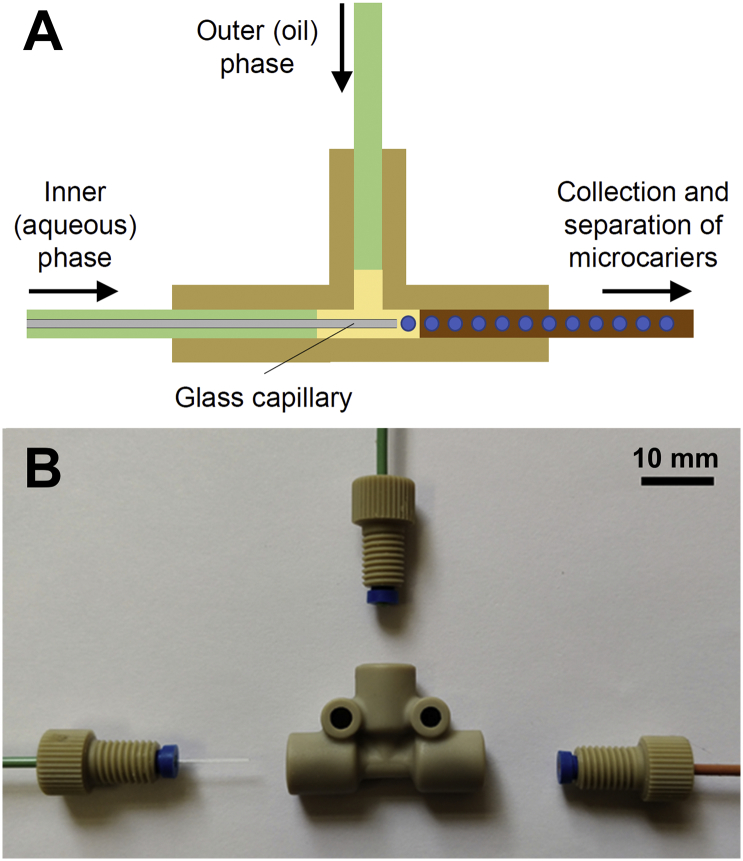
Schematic image (A) and exploded view (B) of the “off-the-shelf” flow focussing device used to generate SF/G microcarriers.

Following the positive 2D results, we investigated microcarriers for the 3D culture and osteogenic differentiation of MSCs. Previous studies have demonstrated the formation of SF microcarriers from simple homogenized emulsions, but this requires volatile organic solvents and results in microcarriers with a wide size distribution [61]. At the size range of interest for cell microcarriers (~300 μm), variations in diameter have a marked effect on the curvature of the particle surface [62], and cells cultured on microcarriers with a wide size distribution therefore experience decidedly different environments. In order to reproducibly produce microcarriers of a narrow, controlled size range, we employed microfluidic flow focussing to produce SF/G microcarriers. The device used was similar to that described by Terray & Hart [27] but with the advantage that no alterations to the re-purposed HPLC fittings were required (Fig. 3). This system was also advantageous in comparison to one-piece 3D printed devices described elsewhere [33,63], in that it could be fully disassembled for cleaning the component parts should any of the channels become blocked.

**Fig. 4:**
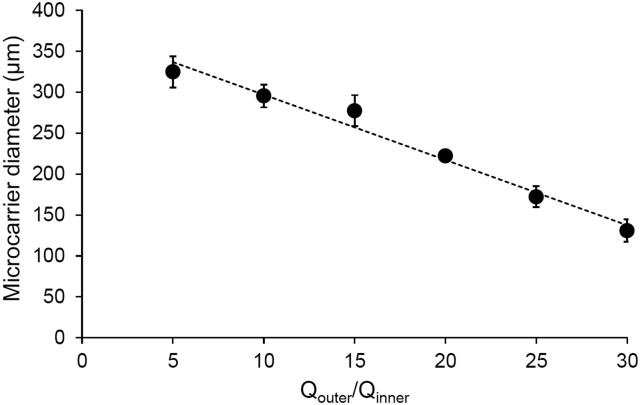
Mean diameter (±standard error) of SF/G 75:25 microcarriers produced under selected flow rate ratios, when Q_inner_ = 0.05 mL/min.

Using an outer phase of oleic acid/methanol/Span 80 (73:25:2) and an inner phase of SF/G solution, microcarriers were produced successfully from SF/G 75:25, SF/G 50:50, and SF/G 25:75. Methanol was employed in the outer phase to induce a conformational change in silk fibroin, resulting in the formation of insoluble crystalline β-sheets which act as physical crosslinks in the protein structure [64]. This effect was combined with cooling the outflow to induce the temperature-dependent gelation of gelatin, allowing the collection of the resultant droplets as microcarriers. It was possible to tailor the diameter of the microcarriers by adjusting the flow rates of the outer and inner phases, and the ratio between the two (Q_outer_/Q_inner_; Fig. 4). Based on preliminary investigations, a Q_inner_ of 0.36 mL/h was the optimum for the consistent generation and collection of microcarriers, with Q_outer_/Q_inner_ of 20 consistently yielding microcarriers with diameters in the desired range of 300–400 μm.

### 3.4 Characterisation of microcarriers

**Fig. 5:**
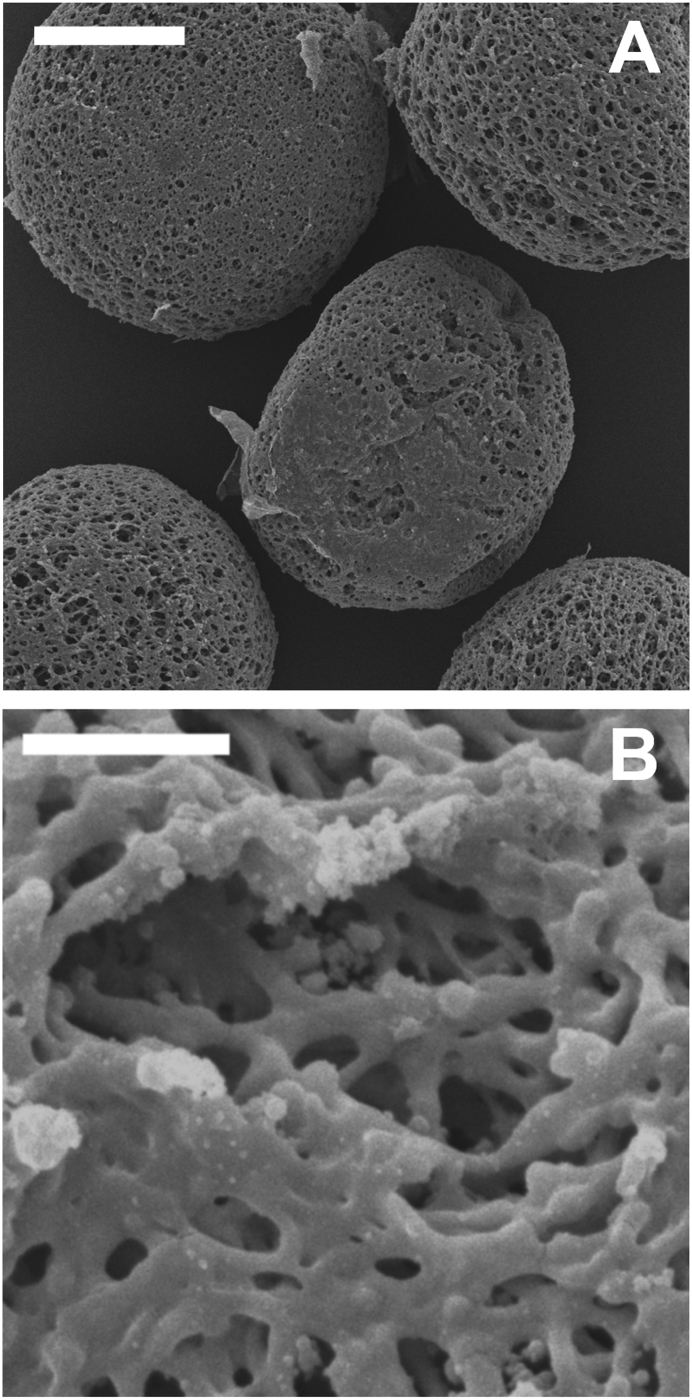
SEM images of SF/G 75:25 microcarriers. The structure observed here was maintained in other SF/G blended microcarriers. Scale bars represent 50 μm (A) and 5 μm (B).

The morphology of the microcarriers was assessed by visible light and scanning electron microscopy. Microcarriers of all three SF/G blends shared a very similar porous surface structure, with representative images of SF/G 75:25 microcarriers shown in Fig. 5. The pores were in the region of ~2 μm, too small for cell migration into the particle interior. Pure SF microcarriers, examined alongside SF/G for comparison, collapsed during lyophilisation, suggesting that the inclusion of gelatin offers improved structural characteristics compared to the non-blended material.

**Fig. 6:**
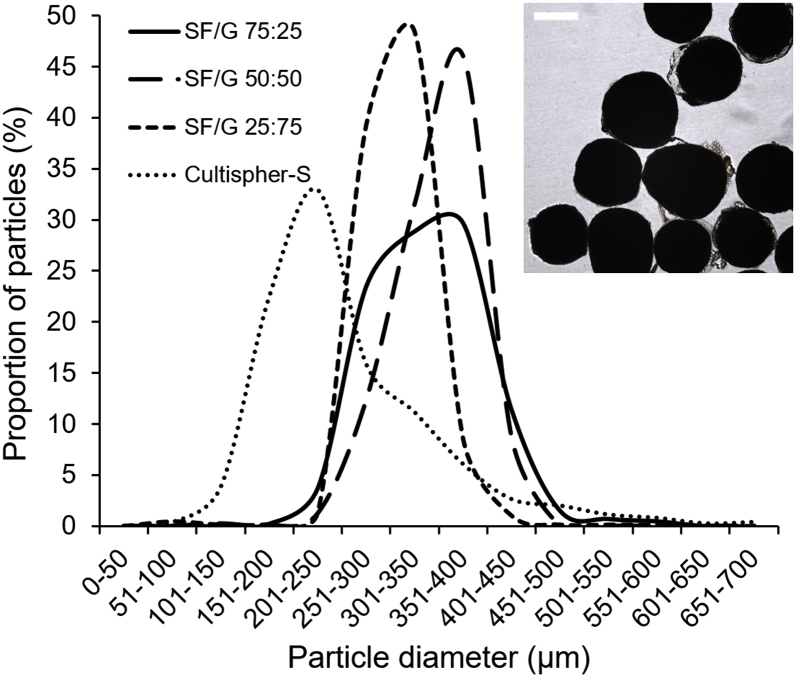
Mean size distributions of SF/G microcarriers produced by microfluidic flow focussing (Q_outer_/Q_inner_ = 20, Q_inner_ = 0.36 mL/h) in comparison to commercial Cultispher-S microcarriers (*n* = 3). Inset: Typical bright field image of SF/G 25:75 microcarriers. Scale bar represents 200 μm.

Microcarrier diameters were measured using ImageJ analysis of visible light microscopy images, and these results are summarized in Table 2 and Fig. 6. The mean diameters of all three blends fell within the target range of 300–400 μm and were comparable with commercially available Cultispher-S gelatin microcarriers. However, in comparison to Cultispher-S, the SF/G microcarriers had a narrower size distribution, especially the SF/G 50:50 and SF/G 25:75 blends, demonstrating the reproducibility and relative homogeneity of this flow focussing approach. Interestingly, product literature for the Cultispher-S microcarriers states a size range of 130–380 μm, although the range measured in this study, 108–565 μm, was considerably larger [65]. To our knowledge, this is the first report of homogeneous SF/G microcarriers. Indeed, there are very few studies in the literature on any silk-based microcarriers, with most SF particle research focusing on either microparticles in the low micron range, or nanoparticles. The only comparable report of SF/G microcarriers describes the cryofragmentation of 3D SF/G scaffolds to generate irregularly-shaped particles with a broad size range of 50 to 300 μm [17]. In terms of pure SF microcarriers, there is one report of a controlled droplet dissolution method to generate transparent silk spheres [66]. These particles are homogenous and, although the paper does not state their size, they appear to be approximately 600 μm in diameter, which is larger than ideal for a cell microcarrier. Other reports of silk-containing microcarriers employ SF as a coating rather than a structural components of the particles. To date, silk-coated pullanan [67] and alginate [68] microcarriers have been described, with diameters of 170 ± 45 μm and 422 ± 46 μm respectively. The particles described in the current study have a narrower size distribution than either of these silk-coated carrier approaches and, as they use SF/G as the matrix of the microcarriers rather than a gel, they are more robust.

**Table 2:** Mean diameters and diameter ranges for SF/G microcarriers produced by microfluidic flow focussing (Q_outer_/Q_inner_ = 20, Q_inner_ = 0.36 mL/h). Values measured for Cultispher-S microcarriers are included for comparison. Data represent the mean ± standard error (*n* = 3).

Material	Particle diameter (μm)	Diameter range (μm)
SF/G 75:25	342 ± 33	201–490 (98.6%)
SF/G 50:50	349 ± 8	213–446 (99.8%)
SF/G 25:75	308 ± 3	218–428 (98.6%)
Cultispher-S	258 ± 11	108–565 (98.6%)

**Fig. 7:**
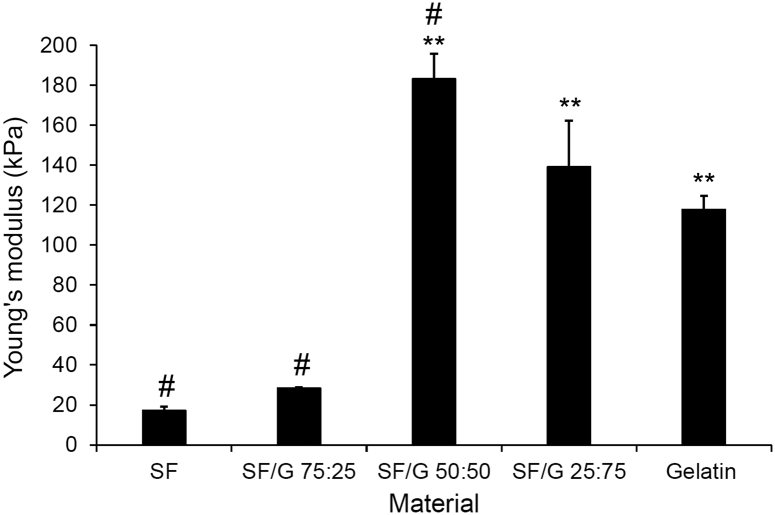
Young's modulus of SF/G gel discs determined by compression testing. Data shown represents mean + standard error (n = 3). ** p < 0.01 w.r.t SF; # p < 0.05 w.r.t gelatin

To examine the mechanical properties of these blends, the Young's modulus of SF/G discs was measured by compression testing and compared to SF and gelatin alone (Fig. 7). Although it would have been preferable to measure the compressive strength of individual microcarriers, their dimensions precluded direct measurement and so the bulk material properties of macro scaffolds were assessed. SF alone was shown to have a relatively low Young's modulus of 17 kPa and, with the inclusion of 25% gelatin, this showed a marginal increase to 28 kPa. The blends of SF/G 50:50 and SF/G 25:75 had significantly higher Young's moduli of 183 kPa and 139 kPa, respectively, while gelatin measured 118 kPa. The compressive strength of scaffolds is influenced by a number of factors, including their microstructure and component materials. It has been reported that SF scaffolds fabricated by gas foaming, salt leaching and freeze drying have compressive moduli ranges of 200–1000, 100–790 and 10–220 kPa respectively [69]. The matrices described here are most similar to the freeze dried scaffolds, with compressive moduli in the same range. Literature reports describe the compressive moduli of lyophilized SF/G scaffolds in the region of 60–160 kPa [70]. The higher value for SF/G 50:50 may be attributed to this blend ratio perhaps producing a more homogenous pore structure than the other blends, contributing to a stiffer material [70]. This again demonstrates the benefits of combining SF and gelatin, as the SF/G 50:50 and 25:75 blends outperform the unblended materials in terms of compressive strength. It is well known that the Young's modulus can have a strong effect on cell behaviour, especially the differentiation of mesenchymal and other stem cells. For an osteogenic scaffold, a higher Young's modulus that mimics the rigidity of bone is desirable. It was reported by Engler et al. that a Young's modulus in the region of 30 kPa was stiff enough to induce the osteodifferentiation of MSCs [71]. Although this is a lower value than that of bone itself, it relates to the osteoid region of bone where MSCs initially differentiate into pre-osteoblasts [72]. The SF and SF/G 75:25 discs have Young's moduli just below this, but the SF/G 50:50 and 25:75 blends were stiff enough to theoretically support osteogenic differentiation according to these criteria. It is also possible that a different range of values could be obtained by altering the cross-linking protocol of the gelatin component of the materials, with higher levels of cross-linking resulting in stiffer materials and *vice versa*.

### 3.5 Cell adhesion and viability on SF/G blended microcarriers

**Fig. 8:**
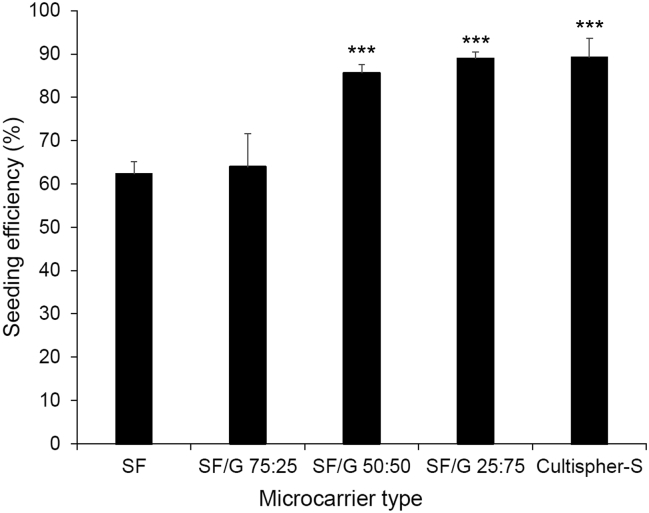
Seeding efficiency (percentage of rMSCs seeded that have adhered to microcarriers) of SF, SF/G and Cultispher-S microcarriers. Data shown represents mean + standard error (*n* = 3). *** *p* < 0.001 w.r.t. SF and SF/G 75:25.

Cell adhesion to the microcarriers, shown in Fig. 8, mirrored the results observed in 2D culture (Fig. 1). Low cell adhesion was observed on the SF microcarriers, while significantly higher seeding efficiencies were obtained with the inclusion of 50 or 75% gelatin, with these blends supporting cell adhesion at a level equivalent to that observed for Cultispher-S microcarriers, included as a positive control. However, for SF/G 75:25 the efficiency was significantly lower, suggesting a general trend of increasing cell adhesion in line with increasing proportion of gelatin, which is in contrast to the pattern of cell proliferation on the 2D films of SF/G, where all gelatin-containing substrates supported high growth rates. The seeding efficiencies can be grouped into moderate and high pairs: SF and SF/G 75:25 are moderate at 62.3% and 63.9%, respectively, while SF/G 50:50 and SF/G 25:75 show higher efficiencies at 85.7% and 88.9%. This grouping reflects the pattern of Young's moduli shown in Fig. 7, and the level of cell adhesion could be directly related to the stiffness of the material blend; it is reported that fibroblastic cells adhere and spread preferentially on stiffer materials [73]. The Young's modulus of the Cultispher-S microcarriers is unknown, but the data here suggests the gelatin matrix is more highly cross-linked than the level of the gelatin films described in this work, producing a stiffer matrix which supports a high level of rMSC adhesion.

### 3.6 Viability and osteogenic differentiation of rMSCs on SF/G microcarriers

**Fig. 9:**
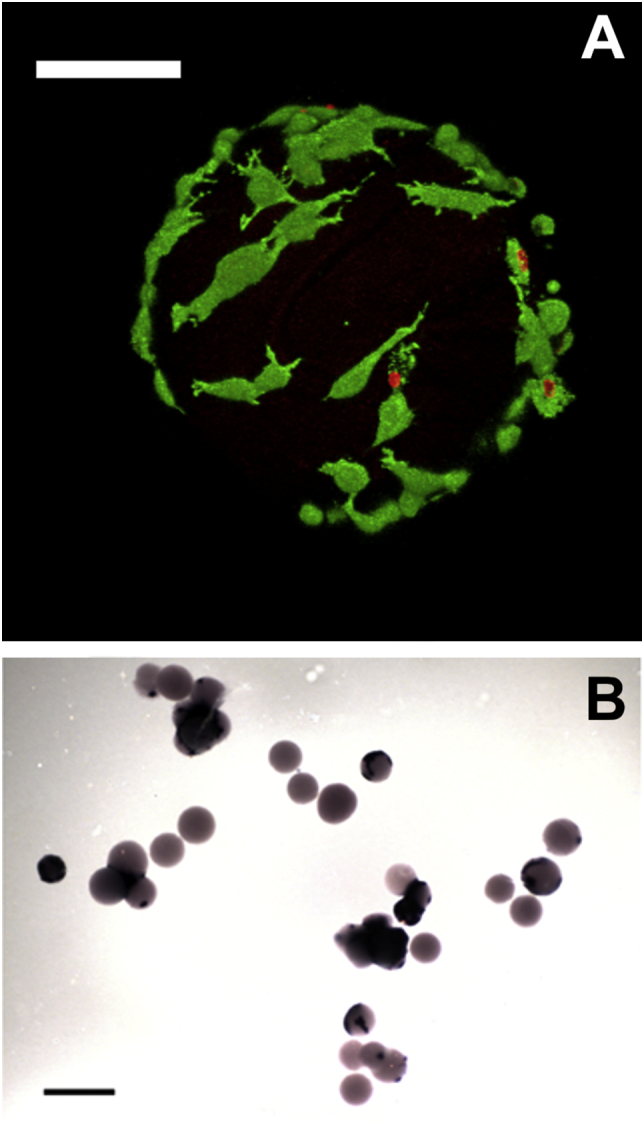
A. Confocal microscopy image of rMSCs seeded onto SF/G 25:75 microcarriers and cultured for 96 h. Cells are stained with LIVE/DEAD to indicate viability. Cells stained green are viable, while cells stained red are dead. Scale bar represents 100 μm. B. SF/G 25:75 microcarriers seeded with rMSCs and stained for alkaline phosphatase activity after 28 days in osteogenic medium. Purple deposits indicate the presence of osteoblasts and, hence, the osteogenic differentiation of rMSCs. Scale bar represents 500 μm. (For interpretation of the references to colour in this figure legend, the reader is referred to the web version of this article.)

**Fig. 10:**
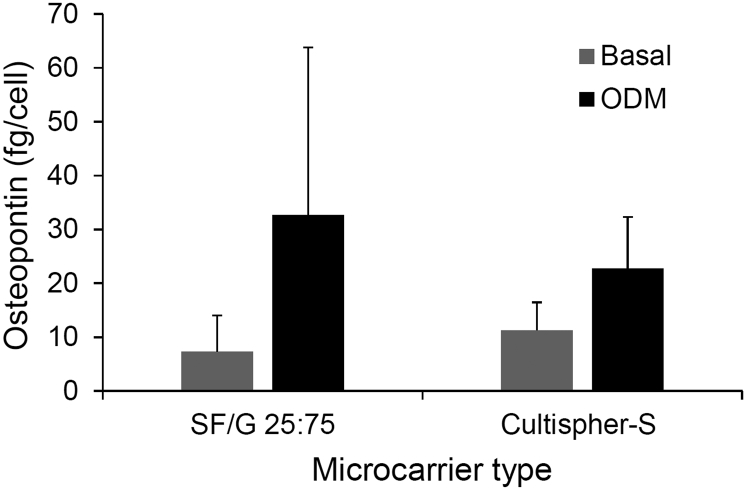
Expression of osteopontin at day 14 in cultures of rMSCs seeded on SF/G 25:75 and Cultispher-S microcarriers, normalized by cell number. Data shown represents mean + standard error (*n* = 5).

For applications in bone tissue engineering, it is important that these microcarriers are able to support not only cell adhesion, but growth, viability and osteogenesis. Viability of rMSCs was confirmed by live/dead fluorescent staining and confocal microscopy of cell-seeded microcarriers. The image in Fig. 9A shows that the cells maintained a high level of viability and were able to spread over the microcarrier surface. The ability of these cells to subsequently undergo osteogenic differentiation was assessed by measuring osteopontin secretion after 14 days in culture. The results of osteopontin expression from rMSCs cultured on either SF/G 25:75 microcarriers (the blend with the best combination of cell and adhesion and elastic modulus) or gelatin-based Cultispher-S microcarriers are shown in Fig. 10. Osteopontin secretion from rMSCs cultured on the SF/G blend was slightly higher than for the Cultispher-S cultured population, although the standard error is quite large, and, as expected, was increased when the cells were cultured in ODM in comparison to the basal medium. These results were also consistent with those from 2D film culture, with a similar quantity of OPN secreted per rMSC in 3D microcarrier culture. A further assessment of osteogenesis was made after 28 days in ODM, when cells were stained for alkaline phosphatase (ALP) expression. In Fig. 9B, rMSCs grown on SF/G 25:75 are shown to have ALP activity, in the form of deep purple staining, which strongly suggests the cells have differentiated to osteoblasts. Although ALP is not uniquely expressed in osteoblasts, it is not expressed in undifferentiated MSCs and so its activity here indicates osteogenic differentiation has occurred. However, the incomplete purple staining in this image does highlight that cell seeding in microcarrier culture isn't always homogeneous and cell-free particles do exist. Nonetheless, these results are in agreement with a number of literature reports describing the successful osteodifferentiation of MSCs on SF-based scaffolds, including SF/chitosan blends [20], SF/G coated decellularized bone [74], and in SF/G bio-ink [75]. To the best of the authors' knowledge, however, this is the first report of SF/G microcarriers produced and applied to this end.

## 4 Conclusions

The results obtained in this study support the use of SF/G microcarriers as scaffolds for cell culture and their development towards bone repair applications. rMSC compatibility and proliferation on silk fibroin and gelatin materials was first confirmed in a two-dimensional format before moving to microcarrier culture. Although silk fibroin alone was shown to have poor cell adhesion, blending silk with gelatin improved the cell adhesion, proliferation and elastic modulus. The SF/G microcarriers were also shown to support cell adhesion and viability as well as osteogenic differentiation of rMSCs. The poor compatibility of the unblended SF and gelatin films highlights the importance of scaffold design for cell response. These microcarriers have the potential to be used as an injectable delivery system for therapeutic cells or a filler material which could stimulate the proliferation and differentiation of resident MSCs for the repair of small bone defects, where load-bearing ability is not a prerequisite. An additional advantage of carrier-based systems is the capacity to scale up the production of therapeutic cells prior to delivery using a stirred, fluidized or rotating bioreactor. Alternatively, the use of the microcarriers as building blocks could allow the development of a macroscopic tissue construct, by moulding a number of cell-laden microcarriers into a fixed architecture for the generation of a number of engineered tissues.
